# The Home Use of Probiotics and Paraprobiotics for the Maintenance of Tongue Eubiosis: A Case Report

**DOI:** 10.1155/crid/5496240

**Published:** 2025-02-17

**Authors:** Salvatore Cannizzaro, Carolina Maiorani, Andrea Scribante, Andrea Butera

**Affiliations:** ^1^Academy of Advanced Technologies in Oral Hygiene Sciences (ATASIO), Siracusa, Italy; ^2^Unit of Dental Hygiene, Section of Dentistry, Department of Clinical, Surgical, Diagnostic and Pediatric Sciences, University of Pavia, Pavia, Italy; ^3^Unit of Orthodontics and Pediatric Dentistry, Section of Dentistry, Department of Clinical, Surgical, Diagnostic and Pediatric Sciences, University of Pavia, Pavia, Italy

**Keywords:** case report, dental hygiene, halitosis, oral eubiosis, paraprobiotics, tongue

## Abstract

**Aim:** Halitosis is the unpleasant odor emitted from the oral cavity during exhalation and phonation. Using oral care products containing specific probiotics and paraprobiotics, combined with lifestyle changes, this study is aimed at resolving the patient's altered breath odor.

**Case Report:** A 49-year-old male patient suffered from retronasal discharge, bacterial plaque on the back of his tongue, and bad breath. He underwent a professional oral hygiene session, received instructions in proper home oral hygiene techniques, and was advised on appropriate lifestyle changes. The use of oral care products based on probiotics and paraprobiotics was recommended. Sixty days later, the patient was reevaluated and was satisfied with the results, as his tongue had improved significantly, and he no longer experienced any bad sensations in his mouth.

**Conclusion:** Systematic removal of bacterial biofilm and the use of probiotics and paraprobiotics can be useful in the prevention and treatment of halitosis.

## 1. Introduction

Halitosis, more commonly known as bad breath, refers to the unpleasant odor of air emitted from the oral cavity during exhalation and phonation [1, 2].

It is mainly due to putrefactive bacteria on the back of the tongue and volatile sulfur compounds (VSCs) produced by food residues: hydrogen sulfide, methylmercaptan and dimethyl sulfide [1–4].

Dry mouth, smoking, eating habits, and alcohol consumption are considered risk factors that can lead to the onset of the problem. Dry mouth, due to reduced salivary flow, encourages anaerobic putrefaction of food residues. Consequently, the bacterial load of Gram-negative bacteria is increased, thereby increasing the production of VSC. Smoking is also often linked to the onset of halitosis as it is linked to high levels of VSC present in tobacco and predisposes to hyposalivation. Certain foods containing volatile compounds can cause an unpleasant oral odor, as can alcohol consumption, due to the oxidation of alcohol in the mouth and liver, producing acetaldehyde and other odorous products; this may also cause hyposalivation and dry mouth [2–4].

Halitosis is classified as either primary, sometimes referred to as extraoral, originating from the lung exhalation, or secondary, sometimes referred to as intraoral, originating from the mouth or upper airway. Most cases of halitosis are secondary, due to VSCs produced in the oral cavity [2–5].

In primary halitosis, fetor hepaticus is one of the main sources for methylmercaptan, particularly when combined with other factors such as smoking, periodontal disease, or xerostomia. Other systemic diseases, not only those affecting the liver but also diseases affecting the respiratory tract, gastrointestinal tract, blood system, and endocrine system, fall into this category. For secondary halitosis, on the other hand, tongue coating, due to the presence of aerobic bacteria on the back of the tongue, and periodontal disease are the main influences. The link between periodontal disease and halitosis can be explained by the decrease in oxygen tension at the level of periodontal pockets, with a concomitant decrease in pH, which is necessary for the putrefaction of amino acids that create VSCs. Another theory supporting the connection between the two problems stems from the fact that periodontitis patients have a significantly increased tongue coating [4–7].

Halitosis is the most frequent cause of dental treatment demand, followed by caries and periodontal disease. Recent studies have estimated the prevalence of halitosis to be between 24% and 39% [6].

The mouth serves not only for chewing or breathing function but also as an organ of communication that attracts the attention of the people around us. Often, patients who are aware of halitosis can feel a real psychological distress. This is why it is preferable for practitioners to use the term “breath odor,” which removes some of the stigma and taboo associated with the term “halitosis” [1].

Studies have shown that some recently introduced compounds could have a significant influence on oral environment [8–10]. The use of postbiotics [11, 12], lysates [13, 14], and paraprobiotics [15] can modify clinical and microbiological parameters in periodontal patients and could be considered as adjuvants for oral odor control [16, 17].

Therefore, the aim of the present report is to combine oral hygiene treatment with the use of probiotics in order to test their combined effect in the treatment of halitosis.

## 2. Case Report

### 2.1. Diagnosis and Etiology

A 49-year-old male patient presented for a professional oral hygiene session. During the objective examination, a patina was noted on the back of his tongue, and the patient informed the doctor of his discomfort due to a perceived unpleasant oral odor following antibiotic therapy.

His medical history showed that he underwent surgery for a deviation of the nasal septum and nasal turbinates, and also confirmed that he suffered from retronasal discharge. In people with rigorous oral hygiene, good dentition and a healthy periodontium, the main cause of halitosis is easily found in the back of the tongue where dental hygienist find yellow mucus from the postnasal drip. This drip is one of the most common causes of halitosis, and its accumulation is influenced by lingual topography. A coating of only 0.1 to 0.2 mm is sufficient to create an oxygen-depleted environment, thus allowing the bacteria that cause bad breath to proliferate. It was then decided to interview the patient to investigate his or her eating habits, lifestyle, and systemic diseases.

An interview with the patient revealed that he was a heavy coffee drinker(about five cups per day). Additionally patient reported to drink little water during the day. Finally, patient reported to wear an FFP2 protective mask at work, which caused his mouth to become drier.

### 2.2. Treatment Objectives

Through the use of oral care products containing specific probiotics and paraprobiotics, in combination with lifestyle changes, this study reported a resolution of the patient's altered breath odor. The results were measured by assessing the level of the lingual patina, the photographic status, and the organoleptic test.

### 2.3. Treatment

The patient showed good dentition, good oral hygiene, and a healthy periodontium, while the tongue appeared neglected by home oral hygiene maneuvers.

The patient's tongue was photographed and images were shared with him, explaining that 90% of altered breath odor cases originate in the mouth. The surface of the tongue, due to its anatomy, is frequently prone to favor deposites of food debris, forming a suitable habitat for the proliferation of anaerobic bacteria that metabolize proteins and produce VSCs. Lingual patina accounts for 60% of oral causes of halitosis; and for those suffering from postnasal drip, this figure is further aggravated.

**Figure 1:**
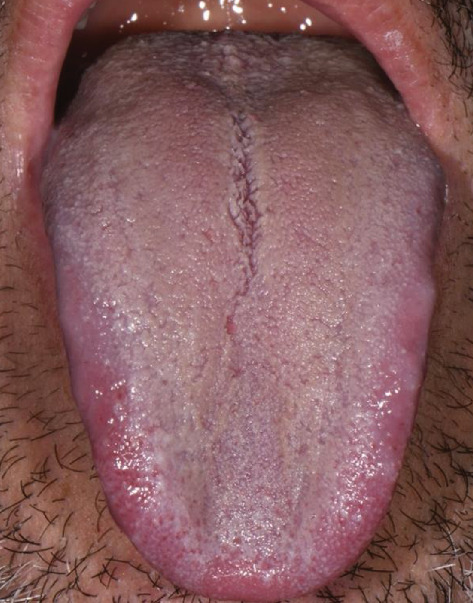
Patient's tongue at T0.

From the topographical spread of the lingual patina on the dorsum of the tongue (Table 1), it was seen to be Grade 2 [18]. In fact (Figure 1), almost the entire dorsum of the tongue was patinated, leaving only a few pink areas; in addition, the anterior third showed mild inflammation at the edges (T0).

It was decided, together with the patient, to carry out an organoleptic assessment of breath odor. To ensure accurate results, it was essential that the patient be examined under standard conditions. The practitioner explained how the patient should prepare for the next appointment. The patient was instructed to

• Come in the morning;
• Not perform oral hygiene maneuvers;
• Fast on food, drinks, chewing gums, or candies;
• Not eat garlic, onions, spices, or halitogenic foods in general, at least 48 h before the examination;
• Abstain from using cosmetics and perfumes on the same day as the visit.

The examination was conducted in a neutral, odor-free room, maintained at a controlled temperature, and with adequate ventilation to avoid external influences on the evaluation. The patient returned to undergo the organoleptic test, which is performed using a plastic tube inserted into the patient's mouth, which prevents the breath from diluting with the air in the chamber. The patient slowly exhaled, and in the meantime, the practitioner judged the odor coming out of the tube by noting it as Grade 3 “moderate odor” [19].

The nasal breath odor is measured by means of a tube inserted into one of the nostrils, while the other nostril is closed. Again, Grade 3 as “moderate odor” is noted.

To facilitate the diagnosis of halitosis, four different parameters were assessed:

• The air emitted from the mouth during phonation (count-to-twenty test) [20];
• The odor produced by the biofilm of the front part of the tongue (wrist lick test) [21];
• The odor produced by the biofilm of the back of the tongue (spoon test) [22];
• The odor generated by microorganisms in the interdental spaces by flossing (floss test) [23].

The count-to-twenty test allowed the observation of breath odor during dialogue. In this case, the patient counted aloud to 20, and the practitioner, positioned 10 cm from his mouth, detected the odor and noted down the number uttered at the moment the odor was perceived. The number noted down by the professional was seven.

The wrist-lick test allowed the smell of the biofilm in the anterior third of the tongue to be observed. The patient was asked to lick his wrist, and after 5 s, the odor was assessed by sniffing at a distance of 5 cm. In this case, Grade 2 “slight but appreciable odor” was noted.

The spoon test was performed using a disposable spoon with which the biofilm of the posterior third of the tongue was taken. Once taken, the biofilm was incubated for 5 s and professional assessed its odor at a distance of 5 cm. The perceived odor grade was 4 “strong odor.”

The floss test was performed by flossing between the back teeth. The odor was assessed after 5 s at a distance of 5 cm from the nose. In this case, the noted grade was 2 “slight but appreciable odor.”

From the results obtained from the various organoleptic tests, it was possible to make a clinical judgment on both the nature of halitosis and the health conditions of the various oral areas and their contribution to the patient's halitosis (Table 2). The diagnosis was Grade 3 “moderate odor” halitosis, with a postnasal drip etiology aggravated by medication [19].

At this point, the debridement and deplaquing session was carried out. Next, the TS1 Zungen Sauger professional tongue cleaner (Ideco) was mounted on the suction canula of the dental unit for professional tongue hygiene. A 12% chlorhexidine gel was applied to the tongue and, using a brush placed on the TS1 Zungen Sauger, was spread over the entire back. Subsequently, the tongue cleaner was turned over, and the bacterial biofilm on the surface was aspirated.

The use of a toothpaste with paraprobiotics was suggested (Peribioma Pro Gengive Più, Coswell, Fano, Italy), to help rebalance the normal oral microbiota, with an antibacterial toothbrush with medium bristles and highly ergonomic, with a curved handle that conforms to the anatomy of the oral cavity (Table 3), allowing it to reach deeper into the mouth (Toothbrush Total Protection, Coswell, Fano, Italy). This toothbrush had bristles made of bacteriostatic material that counteracted bacterial proliferation, while the back featured a soft rubber tongue cleaner. With regard to the brushing technique, it was decided not to make any changes as the patient showed good dental hygiene, but the importance of daily tongue hygiene and its central role in altering breath odor was explained.

The use of antibacterial interdental brushes is also recommended, with their bendable tip allowing the patient to reach the posterior areas more easily (Cylindrical Interdental Pick Ultra Fine).

For complete oral hygiene, it was recommended to use a mouthwash enriched with undiluted zinc PCA and paraprobiotics for at least 1 min, gargling directly on the posterior third of the back of the tongue, without rinsing after use and without eating/drinking for at least 30 min after treatment (antibacterial mouthwash plus with intensive treatment, Coswell, Fano, Italy).

Finally, the use of chewing gum once a day for about 20 min was recommended (chewing gum with Peribioma Pro, Coswell, Fano, Italy). These gums contained probiotics that stimulated immune action and promoted rebalancing of the oral microbiota, also prompting increased salivary production at times when the patient perceived dryness of the mouth.

It was also suggested to reduce coffee consumption, as it is a xerogenic drink. It was suggested not to exceed two cups per day, followed by tooyh brushing. Finally, the importance of adequate water intake was explained, in order to maintain the oral cavity as moist as possible (T0).

### 2.4. Results

**Figure 2:**
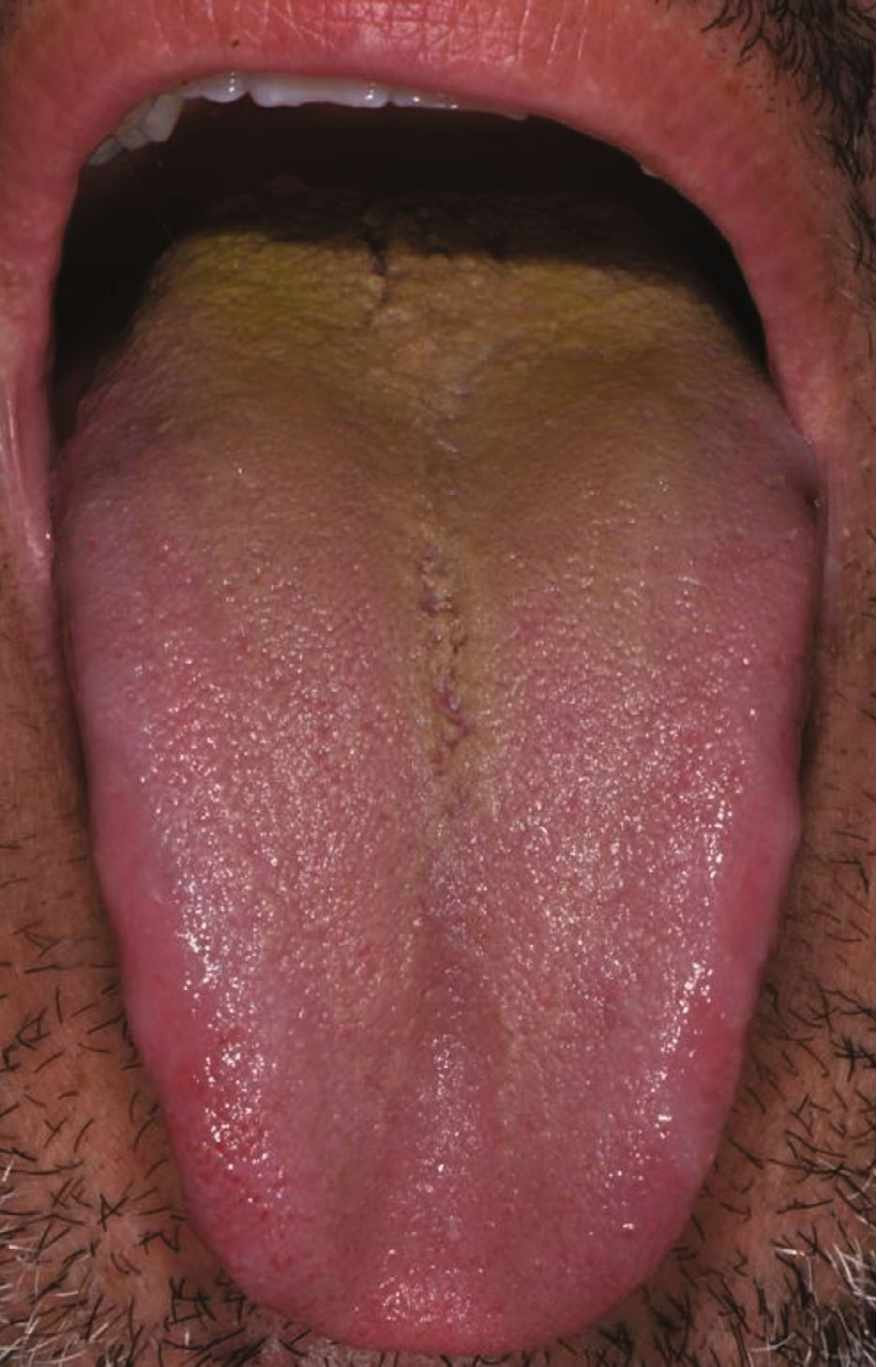
Patient's tongue at T1.

After 21 days, the patient was reevaluated for treatment feedback (Figure 2). Immediately, it was observed that the patina, which almost completely covered the dorsum of the tongue, had disappeared. However, the patina due to the retronasal discharge still persisted on the posterior third. This patina certainly appeared to be more resistant also because it was self-feeding. The patient also confessed to having difficulty in proceeding with such deep maneuvers. He was therefore remotivated and, observing together the improvements he was achieving, he was asked to insist more with the tongue cleaner on the posterior third of the tongue, always maintaining the gentleness of the movements that he had to repeat from the back towards the front (T1).

**Figure 3:**
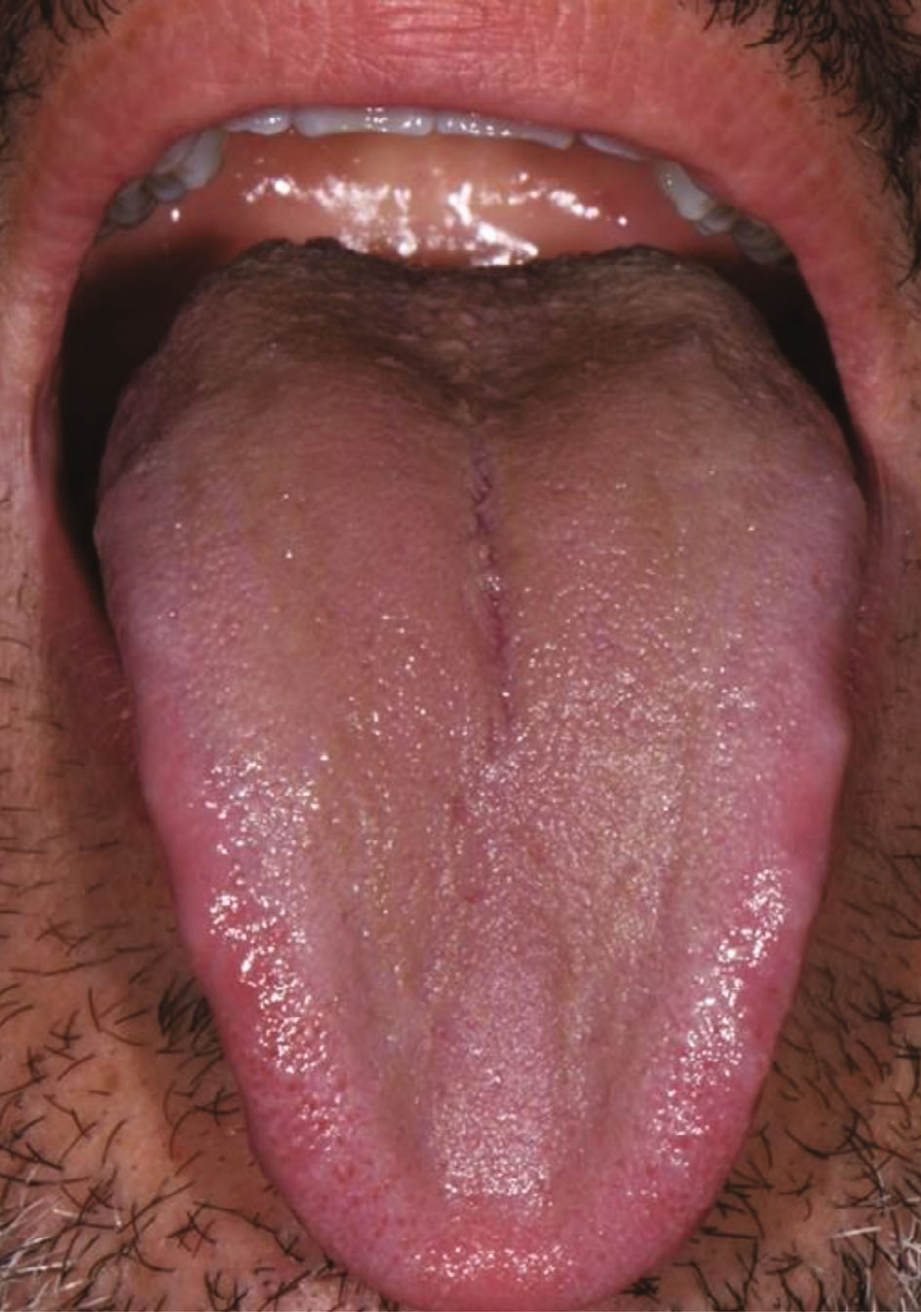
Patient's tongue at T2.

Sixty days later, we see the patient again, and he immediately informs us that he is satisfied with the results obtained, that his tongue has clearly improved and that he no longer feels that bad sensation in his mouth (Figure 3). He also states that he has reduced his coffee consumption and drinks a lot more water during school days; not having to wear the FFP2 protective mask every day due to the arrival of the summer holidays has also brought him relief.

An image of the patient's tongue was taken. The image shows how the lingual patina has disappeared completely. The dental hygienist can then state that the level of lingual patina is at Grade 0 with “no lingual patina.”

The organoleptic assessment was carried out again under standard conditions, and it was observed that the degree of severity of halitosis had improved from Grade 3 to Grade 0 with “no appreciable odor”; in the count-to-twenty test, the noted number was 20. The wrist-lick test was Grade 0 “absence of appreciable odor”; the spoon test was 1 “barely perceptible odor”; and the floss test was 0 “absence of appreciable odor.”

Patient was suggested to to continue the use of the above mentioned probiotic-based toothpaste with medium-bristle toothbrush and integrated tongue cleaner in addition to, interdental brushes (size 0.6), and mouthwash once a day, insisting on rinsing the posterior third of the tongue.

## 3. Discussion

Halitosis is the escape of an unpleasant smell from the mouth, which can depend on several factors, such as the quality and amount of saliva (acidic pH and limited presence), the consumption of certain foods and beverages (garlic, onion, cheese, red meat, spices, coffee, and alcoholic beverages), smoking habit, and taking certain medications (ACE inhibitors, antitumor, antiretroviral, sympathomimetic, opioid analgesics, anorexizing, central nervous system stimulators, anxiolytics and sedatives, antiarrhythmics, antidepressants, antidiarrheals, antimaniacals, antimuscarinics, anti-inflammatories, and antiparkinsonian agents) and various oral and/or systemic pathologies (gingivitis or periodontitis, stomatitis, caries, dental prostheses with bacterial plaque, bacterial or mycotic oral infections; sinusitis, tonsillitis, retronasal discharge, bronchitis, pulmonary abscesses, esophageal diverticula, dysphagia, regurgitation, hiatal hernia, pyrosis, diabetes mellitus, chronic kidney failure, and chronic liver failure) [1–3, 24–26]. The patient treated in this study suffered from retronasal discharge and showed the back of the tongue covered with bacterial plaque (Grade 2). After undergoing an oral hygiene session with instruction and motivation in the correct home methods home and after using probiotic products, has improved his situation of oral halitosis.

Probiotics, in fact, have several advantages, such as antimicrobial activity, powerful binding ability, acid pH neutralization, modulation of oxidation–reduction potential, increased immune system function, and reduction of inflammatory procytokines [27]. These combined qualities lead to the improvement of oral cavity disorders and also to the prevention or improvement of oral halitosis [28]. In the prevention and treatment of halitosis, probiotics are proving to be perfectly adaptable to the human oral ecosystem and thus a valuable health aid [29, 30].

Their mechanism of action, linked to their ability to compete with pathogenic microorganisms for sites of adhesion, prevents and treats oral infections, including dental caries, periodontal disease, and halitosis. Halitosis is not a serious disease but may become one [27–29, 31]. Despite the fact that it affects a significant part of the world population, it is still underestimated. Without proper diagnosis and management, halitosis has a significant impact on the quality of life and can have psychological consequences, including social, professional, and emotional limitations [32]. The available studies on the use of probiotics in the treatment of oral halitosis do not draw significant scientific evidence: they have transient effects on organoleptic indices, but do not confirm reductions of VSCs with the use of Lactobacilli strains [33–35].

It is important that patients are aware that most of the causes of halitosis originate in the oral cavity. As a result, a simple and natural approach to evaluation and therapy puts the dental hygienist in the ideal position to respond to an initial consultation for halitosis [36].

Today, thanks to the remarkable research progress, everyone can satisfy their aesthetic desire to improve their breath. In fact, the problem of halitosis has not only aesthetic relevance; its diagnosis and treatment produce significant improvements in health conditions, both of the oral cavity and psychological well-being, allowing him to solve any discomfort of an emotional nature [37, 38].

This study presents some limitations that need to be considered. First, the results are based on the observation of a single clinical case, which limits the generalizability of the conclusions to other populations with different characteristics. Moreover, a control group was not included, making it difficult to compare the effectiveness of the strategies adopted with alternative or standard treatments.

Another aspect to consider is the limited duration of the follow-up, which does not allow for the evaluation of the long-term effects of the proposed therapies. Some measurements, such as the organoleptic analysis, are based on subjective evaluations, which may introduce variability.

Furthermore, individual factors such as the patient's lifestyle and dietary habits may have influenced the results, further limiting the applicability of these conclusions to a broader population. The study also did not analyze in detail the microbiological changes in the oral microbiota or the levels of VSCs, leaving uncertainties about the precise mechanism of action of the probiotics.

Despite its limitations, this study suggests that the systematic removal of bacterial biofilm and the use of probiotics and paraprobiotics can be useful in the prevention and treatment of halitosis. Future studies are needed to establish significant efficacy, also aimed at highlighting the most useful strains and dosages.

## 4. Conclusions

Bad breath is an underestimated problem that affects the world's population. It is an unpleasant odor emanating from the mouth and has a multifactorial etiology. By motivating the patient to perform correct oral hygiene at home with lingual devices and by adding probiotics, which restore the oral microbiota and reduce the incidence of dysbiosis, they can be used both to prevent and to treat oral malodor, improving clinical indices thanks to their action against gram-negative bacteria.

### 4.1. Take Home Message

The systematic removal of bacterial biofilm and the use of probiotics and paraprobiotics, together with oral hygiene, can be useful in the treatment of halitosis.

## Figures and Tables

**Table 1:** Topographical spread of the lingual patina [18].

**Value**	**Description**
0	No visible patina on the back of the tongue
1	Patina present only on the Posterior III of the tongue
2	Patina that completely covers the dorsal surface of the tongue but does not mask the underlying mucosa
3	Very thick patina covering the entire dorsal surface of the tongue

**Table 2:** Organoleptic evaluation (sniff test) [19].

**Value**	**Description**
0	No appreciable odor
1	Barely noticeable odor
2	Slight, but clearly noticeable odor
3	Moderate odor
4	Strong odor
5	Extremely foul odor

**Table 3:** Products used for oral hygiene.

**Product**	**Ingredients**
Toothpaste Peribioma Pro Gengive Più, Coswell SPA, Fano, Italy	Aqua, zinc hydroxyapatite, sorbitol, glycerin, hydrated silica, silica, cocamidopropyl betaine, cellulose gum, zinc PCA, aroma, pistacia lentiscus (mastic) gum oil, ascorbic acid, tocopheryl acetate, retynil palmitate, sodium hyaluronate, *Hamamelis virginiana* leaf extract, spirulina platensis extract, calendula officinalis flower extract, eucaliptus globulus leaf oil, *Bifidobacterium*, *Lactobacillus*, sodium myristoyl sarcosinate, sodium methyl cocoyl taurate, phenoxyethanol, benzyl alcohol, sodium benzoate, sodium saccharin, potassium sorbate, maltodextrin, citric acid, *Helianthus annuus* seed oil, BHT, limonene, eugenol, C.I. 77891, C.I. 73360

Antibacterial mouthwash plus with intensive treatment, Coswell SPA, Fano, Italy	Aqua, sorbitol, zinc PCA, xylitol, cellulose gum, zinc hydroxiapatite⁣^∗^, aroma, lactobacillus lysate (*Lactobacillus rhamnosus* LR06; *Lactobacillus pentosus* LPS01; *Lactobacillus plantarum* LP01; *Lactobacillus delbrueckii* LDD01), lactoferrin, Peg-40 hydrogenated castor oil, sodum lauryl sulfate, sodium benzoate, benzyl alcohol, sodium saccharin, limonene.⁣^∗^microRepair

Chewing gum with Peribioma Pro, Coswell SPA, Fano, Italy	Gum base (flavourings; emulsifier—soya lecithin; sweeteners—acesulfame, sucralose; antioxidant—tocopherols); bulking agents - isomalt, sorbitol; microRepair (calcium salts of orthophosphoric acid); probiotic lactic ferments [*Lactobacillus reuteri* (SGL 01, *Lactobacillus salivarius* (SGL 03), *Lactobacillus plantarum* (SGL 07); support—maltodextrin from corn; anticaking agent—silicon dioxide], flavourings, vitamin C (calcium ascorbate), food colouring substances (radish and sweet potato concentrate); sweeteners—sucralose, acesulfame K; vitamin D (cholecalciferol)

## Data Availability

Data sharing is not applicable to this article as no new data were created or analyzed in this study.
